# Introduction to BLAH5 special issue: recent progress on interoperability of biomedical text mining

**DOI:** 10.5808/GI.2019.17.2.e12

**Published:** 2019-06-27

**Authors:** Jin-Dong Kim, Kevin Bretonnel Cohen, Nigel Collier, Zhiyong Lu, Fabio Rinaldi

**Affiliations:** 1 Database Center for Life Science, Research Organization of Information and Systems, Kashiwa 277-0871, Japan; 2 School of Medicine, University of Colorado, Aurora, CO 80045, USA; 3 Faculty of Modern and Medieval Languages, University of Cambridge, Cambridge CB3 9DP, UK; 4 National Center for Biotechnology Information (NCBI), U.S. National Library of Medicine (NLM), Bethesda, MD 20894, USA; 5 Institute of Computational Linguistics, University of Zurich, Zurich CH-8050, Switzerland; 6 IDSIA, Manno CH-6928, Switzerland; 7 Swiss Institute of Bioinformatics, Lausanne CH-1015, Switzerland

Position papers and keynote speeches have often pointed out the great potential of linked data as a mechanism for unifying biological databases with the biomedical literature, but there have been few long-term and large-scale explorations of the research and technical issues that such a unification would raise. To address that paucity of studies, the Biomedical Linked Annotation Hackathon (BLAH) has met for the past five years in order to bring together experts from a variety of relevant disciplines and to create an environment in which they can collaborate on highly focused projects for a short, intense period of colocation.

BLAH is organized annually by the Database Center for Life Science (DBCLS), Research Organization of Information and Systems (ROIS). The goal of the BLAH series is to enhance the interoperability of resources for biomedical text annotation and mining, which we believe is a key for the next breakthrough of biomedical text mining. This special issue delivers seven application notes and two mini reviews, under the theme, “biomedical text mining.” They are outcomes from the 5th Biomedical Linked Annotation Hackathon (BLAH5), which was held from 12th through 15th February 2019 in Kashiwa, Japan.

The nine papers included in this special issue are the results of mini projects investigated before, during and/or after BLAH5, for which BLAH5 aimed to provide a good motivation and an ideal environment to make breakthroughs through intensive collaboration among various experts participated in the three days of hackathon. The papers represent various recent issues of biomedical text mining, ranging across sharing and interconnecting datasets, tools and platforms of biomedical text annotation, privacy issues, multilingualism, and machine learning. The application domains include, clinical domain, drug repurposing, rice biology, microbial biology, and so on. However, they were all investigated under the interoperability theme during BLAH5.

We hope it to be an opportunity for audience of the journal of *Genomics and Informatics* to be aware of the issues and progress of biomedical text mining from the perspective of interoperability.

